# Gut Microbiota Dysbiosis in Japanese Female Patients with Nontuberculous Mycobacteria-Associated Lung Disease: An Observational Study

**DOI:** 10.3390/biomedicines13051264

**Published:** 2025-05-21

**Authors:** Kanako Kono, Yutaka Kozu, Shun Yokota, Kouta Hatayama, Kenji Mizumura, Shuichiro Maruoka, Hiroaki Masuyama, Yasuhiro Gon

**Affiliations:** 1 Symbiosis Solutions Inc., 2-8-11 Kandasarugakucho, Chiyoda-ku, Tokyo 101-0064, Japan; kono@symbiosis-solutions.co.jp (K.K.); hatayama@symbiosis-solutions.co.jp (K.H.);; 2 Division of Respiratory Medicine, Department of Internal Medicine, Nihon University School of Medicine, 30-1 Ohyaguchi-Kamicho, Itabashi-ku, Tokyo 173-8610, Japan; kozu.yutaka@nihon-u.ac.jp (Y.K.); yokota.shun@nihon-u.ac.jp (S.Y.); mizumura.kenji@nihon-u.ac.jp (K.M.); gon.yasuhiro@nihon-u.ac.jp (Y.G.)

**Keywords:** gut microbiota, microbiome dysbiosis, nontuberculous mycobacteria, nontuberculous mycobacterial pulmonary disease, Japanese female patients, 16S rRNA sequencing

## Abstract

**Background/Objectives**: Nontuberculous mycobacterial pulmonary disease (NTM-PD) is treated using a combination of multiple antimicrobial agents and prolonged therapy; however, recurrence and reinfection rates remain high. Susceptibility to NTM-PD is not fully understood. We aimed to investigate the association between NTM-PD and gut microbiota and determine the impact of antimicrobial therapy on the composition of the gut microbiota. **Methods:** We analyzed the gut microbiota of 20 Japanese females with NTM-PD (mean age: 67.9 years; range: 50–80 years) at different treatment stages—before, during, and at recurrence—alongside 20 healthy individuals, using 16S rRNA gene amplicon sequencing. **Results:** Subgroup A (pre-treatment) showed a small difference in β-diversity when compared with the healthy control (HC) group, while no significant differences in α-diversity were observed. Subgroup B (during treatment) exhibited a larger difference in β-diversity compared with the HC group, along with a decrease in α-diversity. The α-diversity of the gut microbiota in Subgroup C (at recurrence) was lower than that in Subgroup A but higher than that in Subgroup B. In Subgroups A and C, the bacterial taxa *Sutterella*, *Adlercreutzia*, *Odoribacter*, and *Prevotella* had decreased relative abundance, while *Erysipelatoclostridium*, *Massilimicrobiota*, *Flavonifractor*, *Eggerthella*, and *Fusobacterium* had increased relative abundance compared to those in the HC group. **Conclusions:** The loss of normal resident gut bacteria may hinder reacquisition. Treatment may be associated with the persistence of a dysbiotic gut microbiota, fostering susceptibility to NTM-PD. Gut microbiota dysbiosis may heighten susceptibility to NTM-PD, complicate treatment outcomes, and increase the risk of microbiological recurrence following therapy.

## 1. Introduction

Nontuberculous mycobacteria (NTM) refer to mycobacteria other than *Mycobacterium tuberculosis* and *Mycobacterium leprae*. They are ubiquitous bacteria inhabiting natural environments, such as soil and water, and artificial environments like bathrooms [1,2]. The incidence of nontuberculous mycobacterial pulmonary disease (NTM-PD) is increasing worldwide [3] and in Japan, posing a significant public health concern [4]. In Japan, most cases occur in individuals aged 70 and older, with women accounting for 65.5% of cases [5]. Moreover, a nationwide study analyzing Japanese health insurance claim data from 2009 to 2014 showed that 69.6% of newly diagnosed NTM-PD cases were women, with a mean age of 69.3 years [6]. Among Japanese individuals with NTM-PD, *Mycobacterium avium* and *Mycobacterium intracellulare* are the most common NTM species [5]. Managing NTM-PD is challenging and typically requires a combination of multiple antimicrobial agents and prolonged therapy [3,7]. Despite successful completion of antimicrobial treatment, approximately 50% of individuals may experience microbiologic recurrence, primarily due to reinfection rather than relapse [3,8].

Although NTM species are ubiquitous in the environment and most individuals are exposed, infection remains rare, as normal host defense mechanisms typically prevent NTM infection. However, individuals with NTM-PD may be predisposed to developing the disease following exposure [9,10]. Structural lung diseases, such as bronchiectasis and chronic obstructive pulmonary disease, are predisposing factors for NTM-PD; however, the disease can also occur in individuals with no underlying lung conditions [10]. Additionally, NTM-PD is frequently observed in thin, older female patients without apparent immunodeficiency [11]. Since the exact mechanisms underlying susceptibility to NTM-PD remain unclear, the most effective preventive measures have yet to be determined.

Recently, multiple studies have explored the relationship between NTM-PD and the gut microbiota, with findings suggesting that gut microbiota abnormalities may contribute to susceptibility to NTM infection [12,13,14]. A study conducted in Taiwan identified significant dysbiosis in individuals with NTM-PD (NTM-LD, nontuberculous mycobacterial lung disease) and reported that alterations in the gut microbiota could lead to a fundamental decline in immune function, potentially triggering the onset of NTM-PD [13]. A reduction in the abundance of *Prevotella copri* was significantly associated with NTM-PD and disease severity. In mouse studies, oral administration of *P. copri* or its capsular polysaccharides enhanced TLR2 signaling, restored immune responses, and improved susceptibility to NTM-PD. Additionally, a study in South Korea found that gut microbiota composition in individuals with NTM-PD differed from that of healthy individuals, with lower α-diversity observed in the NTM-PD group [14].

Recent studies have increasingly focused on the role of the gut–lung axis in shaping host susceptibility to respiratory infections, including nontuberculous mycobacterial pulmonary disease (NTM-PD). Gut microbiota dysbiosis has been reported in patients with NTM-PD, characterized by reduced microbial diversity and altered taxonomic composition, particularly a decrease in *P. copri* abundance [12,13]. This dysbiotic state has been linked to impaired systemic immune responses, including dysfunction of Toll-like receptor 2 (TLR2) signaling, a critical pathway in mycobacterial recognition and defense [12]. Supporting translational evidence demonstrates that antibiotic-induced gut dysbiosis in murine models increases pulmonary colonization by NTM, an effect that is partially reversed by *P. copri* supplementation. Moreover, fecal microbiota transplantation from dysbiotic mice to germ-free recipients enhances susceptibility to NTM infection, accompanied by upregulated *Nos2* expression in lung epithelial cells [15]. These findings suggest that intestinal hyperpermeability and systemic immune dysregulation—consequences of microbiota imbalance—may facilitate pathogen translocation and exacerbate pulmonary inflammation [12,14]. Taken together, these data highlight the potential of gut microbiota modulation as a novel therapeutic approach in vulnerable populations. However, further research is needed to clarify the causal relationships and define precise intervention strategies.

The gut microbiota of Japanese individuals is known to have a distinct composition compared to that observed in other populations [16]. However, no studies have specifically examined the gut microbiota in Japanese individuals with NTM-PD. This is a critical knowledge gap, as findings from other populations may not be directly applicable to the Japanese population. Addressing this gap may facilitate the development of tailored interventions to manage gut microbiota dysbiosis and improve treatment outcomes for individuals with NTM-PD in Japan.

Moreover, given that microbiologic recurrence is common in individuals with NTM-PD, prolonged antimicrobial therapy may exacerbate gut microbiota dysbiosis, potentially increasing susceptibility to NTM-PD. Therefore, in this study, we investigated the gut microbiota of Japanese female individuals aged 50 to 80 years with NTM-PD at different treatment stages (pre-treatment, during treatment, and recurrence) to explore the relationship between NTM-PD and gut microbiota and to clarify the challenges associated with antimicrobial therapy.

## 2. Materials and Methods

### 2.1. Participants

The participants in this study were recruited from Nihon University Itabashi Hospital (Tokyo, Japan). We included Japanese female patients diagnosed with NTM-PD (recruitment period: September 2021 to February 2024). The diagnosis of NTM-PD was based on the 2020 guidelines of the American Thoracic Society/Infectious Diseases Society of America. Written informed consent was obtained from all participants. All experimental procedures adhered to the principles of the Declaration of Helsinki and were approved by the Ethics Committee of Nihon University Itabashi Hospital (Approval Number: RK-210608-14, Approval Date: 22 June 2021).

The data for the healthy participants were obtained from individuals recruited by Symbiosis Solutions Inc. (Tokyo, Japan) between April 2020 and October 2022. Written informed consent was obtained from all participants. All experimental procedures complied with the principles of the Declaration of Helsinki and were approved by the Institutional Review Board of Shiba Palace Clinic (Tokyo, Japan) (Approval Number: 144131_rn-27593, Approval Date: 9 January 2020; Approval Number: 145968_rn-29327, Approval Date: 12 November 2020). Background information was collected through self-reported questionnaires completed at the time of stool sampling. This information included age, sex, height, weight, pregnancy and breastfeeding status, antimicrobial agent and enema use, and medical history.

#### Inclusion and Exclusion Criteria

For the NTM-PD group, we included Japanese female patients aged 50 to 80 years who were diagnosed with NTM-PD based on the 2020 guidelines of the American Thoracic Society/Infectious Diseases Society of America and who provided written informed consent. For the healthy control (HC) group, we included Japanese individuals who provided written informed consent, were age-matched to the NTM-PD group, and completed background questionnaires at the time of stool sampling. We excluded non-Japanese individuals, pregnant or breastfeeding women, individuals who had used enemas, those who had taken antibiotics within the past 3 months (excluding those treated for NTM-PD), and those with incomplete responses to exclusion-related questions in the questionnaire. Additionally, individuals outside the 50–80 years age range were excluded from the NTM-PD group.

### 2.2. Selection of Participants

From the eligible NTM-PD cohort, 20 female patients aged 50 to 80 years were selected based on the exclusion criteria; they formed the NTM group. The medical history of the patients with NTM-PD is provided in Supplementary Table S1.

Among healthy participants, 20 individuals were randomly sampled to match the age distribution in the NTM group, forming the HC group. Healthy participants were excluded if they were currently ill or undergoing treatment for any medical condition.

The NTM-PD group was further categorized into three subgroups based on treatment status at the time of stool sample collection: Subgroup A (treatment-naïve patients who subsequently initiated antibiotic therapy), Subgroup B (patients undergoing active antibiotic treatment), and Subgroup C (patients with a history of treatment, scheduled to restart therapy due to recurrence).

### 2.3. 16S rRNA Data Analysis

Stool sample collection and DNA extraction were conducted using the method by Hatayama et al. [16]. 16S rRNA gene (variable regions V3–V4) sequencing was performed using the MiSeq system (Illumina, San Diego, CA, USA), following the method by Hatayama et al. [17], except for the use of the primers. DNA was extracted from stool samples using a fully automated enterobacterial DNA extractor (DEX-I; PMT Corporation, Fukuoka, Japan), based on the bead crushing method followed by the phenol–chloroform method. Using DNA extracted from stool specimens, variable regions V3 to V4 of the 16S rRNA gene were amplified using 341F primer (5′-TCGTCGGCAGCGTCAGATGTGTATAAGAGACAGCCTACGGGNGGCWGCAG-3′) and 805R primer (5′-GTCTCGTGGGCTCGGAGATGTGTATAAGAGACAGGACTACHVGGGTATCTAATCC-3′). To prepare a DNA library for Illumina MiSeq sequencing using Nextera XT Index Kit v2 primers (Illumina, San Diego, CA, USA). The purified index PCR product was processed on a MiSeq system (Illumina, San Diego, CA, USA) using Reagent Kit v3 (Illumina, San Diego, CA, USA) for DNA sequencing. Amplicon Sequence Variants (ASVs) were generated using the DADA2 v.1.16.0 package [18] in R software v. 4.0.3 (R Foundation for Statistical Computing, Vienna, Austria) [19]. Taxonomic assignment of ASVs was performed using the Ribosomal Database Project (RDP) training set v. 18 [20] (Available online at: https://zenodo.org/record/4310151#.ZDUBAXbP2Ht; accessed 11 April 2023). The ASV table at the genus level was rarefied based on sequence coverage [21] (gradient: ≤0.002259329) using the vegan v.2.5.7 package in R [22].

### 2.4. Gut Microbiota Analysis

α diversity analysis and inter-group comparisons of the gut microbiota were performed using the ANOVA-like differential expression tool (ALDEx2) [23,24,25], following the method by Hatayama et al. [16]. For ALDEx2 comparisons, center log-ratio transformed gut microbiota data were used. To visualize β diversity, nonmetric multidimensional scaling (NMDS) based on the Bray–Curtis index was applied. NMDS was conducted using the metaMDS function from the vegan v. 2.6-4 package in R v. 4.2.0 [22]. Permutational Multivariate Analysis of Variance (PERMANOVA) was conducted using the adonis function from the vegan v.2.6-4 package with 9999 permutations. Permutational Multivariate Analysis of Dispersion (PERMDISP) (multivariate homogeneity of group dispersions) [26] was performed using the betadisper function from the vegan v.2.6-4 package.

### 2.5. Statistical Analysis

Statistical analysis was conducted using R software v. 4.1.0 or Excel Statistics (BellCurve for Excel v3.23, Social Survey Research Information Co., Ltd., Shinjuku, Japan). The Shapiro–Wilk test was applied to assess the normality of continuous variables before comparison using nonparametric methods. For inter-group comparisons of age and body mass index (BMI), the Wilcoxon rank-sum test was performed. For inter-group comparisons of Bray–Curtis distance and α diversity, the Kruskal–Wallis test and Steel–Dwass test were used. Statistical significance was set at *p* < 0.05 (two-tailed test).

## 3. Results

### 3.1. Demographic Characteristics

The data of Japanese female patients aged 50 to 80 years with NTM-PD were analyzed as the NTM group, while that of healthy Japanese female patients of the same age group was analyzed as the HC group. No significant age difference was observed between the NTM and HC groups (Table 1). However, BMI was significantly lower in the NTM group than in the HC group.

### 3.2. Different Gut Microbiota Composition Between NTM and HC Groups

**Figure 1:**
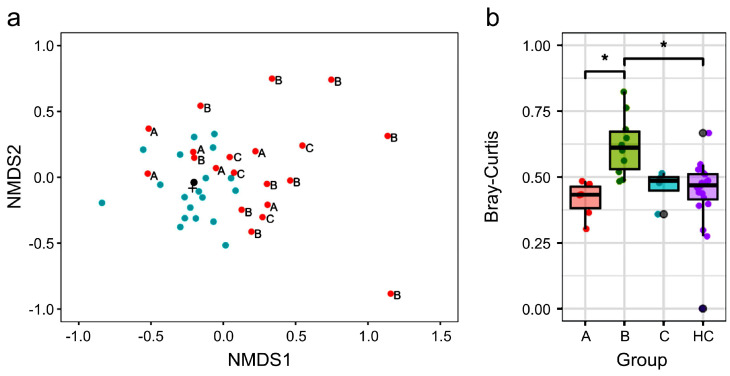
β diversity analysis of gut microbiota in the NTM and HC groups. (**a**) NMDS plot based on Bray–Curtis dissimilarity shows distinct clustering between the NTM and HC groups. Green dots represent samples from the healthy control (HC) group, while red dots represent samples from patients with NTM-PD. (**b**) A boxplot with jitter displays the Bray–Curtis distances of the HC group and each NTM subgroup’s samples relative to HC-cent. The Kruskal–Wallis test showed a significant difference between the four groups (*p* < 0.001). Asterisks indicate significant differences based on the Steel–Dwass test (A vs. B: *p* = 0.008; A vs. C: *p* = 0.551; A vs. HC: *p* = 0.631; B vs. C: *p* = 0.097; B vs. HC: *p* = 0.003; C vs. HC: *p* = 0.988). HC, healthy controls; NTM, nontuberculous mycobacteria; NMDS, nonmetric multidimensional scaling.

To assess the differences in gut microbiota composition between the NTM and HC groups, PERMANOVA was conducted on β diversity. The results indicated a significant difference in β diversity between the two groups (PERMANOVA: *p* = 0.0021, PERMDISP: *p* = 0.115). The NMDS plot visualizing β diversity based on the Bray–Curtis index also demonstrated that the plots of the two groups were positioned differently. The plot of the HC group formed a cluster, suggesting similar gut microbiota composition. Contrarily, numerous plots of the NTM group were positioned away from the clusters of the HC group. The β diversity analysis, including NMDS, suggested that gut microbiota composition differed between the NTM and HC groups (Figure 1a).

### 3.3. Treatment Status and β Diversity of Gut Microbiota in Participants

The NTM group included individuals with different treatment statuses. Therefore, we classified the NTM group into three subgroups based on treatment status: Subgroup A (pre-treatment), Subgroup B (on-treatment), and Subgroup C (pre-treatment with recurrence) (Table 2). The antibiotics used in Subgroup B are shown in Supplementary Table S2. All individuals in Subgroup C had a treatment history with a combination of Rifampicin, Ethambutol, and Clarithromycin, with treatment completion ranging from approximately 6 months to 3 years and 9 months prior. For the NMDS plot, we examined the relationship between treatment status and gut microbiota composition by displaying the information for each NTM subgroup (Figure 1a). Consequently, the plots of participants in the NTM Subgroups A and C, who had not used antibiotics in the past 3 months, tended to be positioned closer to the HC group. Contrarily, the plot for participants in the NTM Subgroup B, who were undergoing antibiotic treatment, tended to be positioned farther from the HC group, suggesting significant differences in gut microbiota composition. This finding was further supported by the differences in the Bray–Curtis distances between the HC-cent sample (the sample closest to the centroid of the HC group plot, as shown in Figure 1a) and each NTM subgroup sample (Figure 1b). When using the HC-cent sample as a reference, the Bray–Curtis distance for NTM Subgroup B was significantly greater than that for Subgroup A and the HC group.

### 3.4. Comparison of α Diversity Between the HC Group and NTM Subgroups

**Figure 2:**
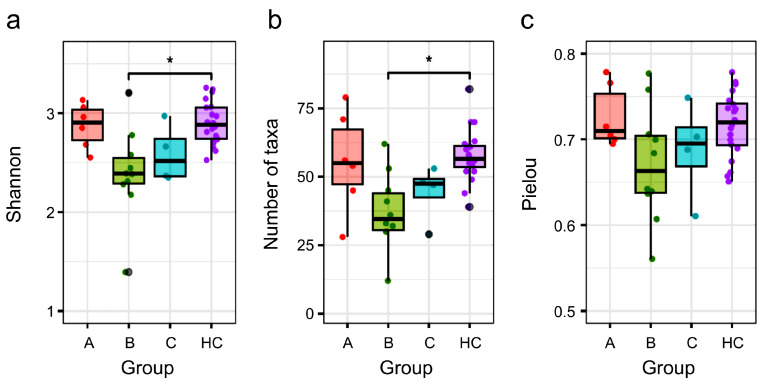
α diversity in HC group and NTM Subgroups A, B, and C. Panels (**a**–**c**) display boxplots with jitter for the Shannon index, number of taxa, and Pielou’s evenness index, respectively. The results of the Kruskal–Wallis test indicated significant differences in the Shannon index and number of taxa across the four groups (*p* < 0.05). Asterisks denote significant differences based on the Steel–Dwass test (*p* < 0.05). The *p*-values of (**a**) are as follows: A vs. B: *p* = 0.093; A vs. C: *p* = 0.416; A vs. HC: *p* = 0.994; B vs. C: *p* = 0.884; B vs. HC: *p* = 0.005; C vs. HC: *p* = 0.226. The *p*-values of (**b**) are as follows: A vs. B: *p* = 0.256; A vs. C: *p* = 0.688; A vs. HC: *p* = 0.989; B vs. C: *p* = 0.851; B vs. HC: *p* = 0.004; C vs. HC: *p* = 0.069. The *p*-values of (**c**) are as follows: A vs. B: *p* = 0.233; A vs. C: *p* = 0.551; A vs. HC: *p* = 0.971; B vs. C: *p* = 0.971; B vs. HC: *p* = 0.179; C vs. HC: *p* = 0.725. Subgroup A refers to individuals with no prior NTM treatment who initiated antibiotic therapy, Subgroup B represents individuals currently undergoing antibiotic treatment, and Subgroup C represents individuals with a treatment history who are about to restart antibiotic therapy. HC, healthy controls; NTM, nontuberculous mycobacteria.

We analyzed the α-diversity of each NTM subgroup with different treatment statuses compared to that in the HC group. Regarding α-diversity indices, we evaluated the Shannon index, which reflects both community richness and evenness—the number of taxa indicating richness and Pielou’s evenness index. The values of the three α-diversity indices for NTM Subgroup A were comparable to those of the HC group (Figure 2). In contrast, the values of the 3 α-diversity indices for NTM Subgroups B and C tended to be lower than those for the HC group. Notably, NTM Subgroup B, which was undergoing antibiotic treatment, exhibited a greater reduction in α-diversity compared to that in the HC group, with significant differences in the Shannon index and the number of taxa observed between the two groups.

### 3.5. Different Gut Bacteria Between HC Group and NTM Subgroups

**Figure 3:**
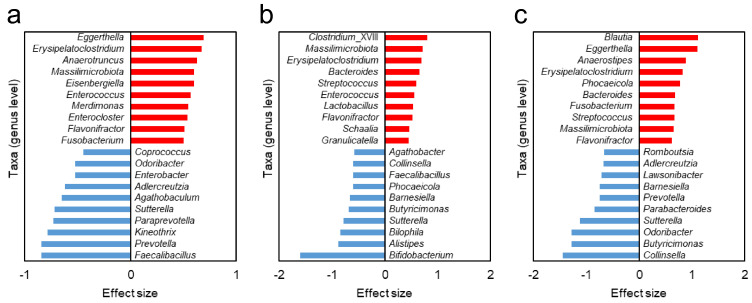
Different bacterial taxa between the HC group and the NTM subgroups. Based on the ALDEx2 analysis comparing the gut microbiota between the HC group and each NTM subgroup, the 10 taxa with the highest and lowest effect sizes (at the genus level) are shown. Panels (**a**–**c**) display the results for NTM Subgroups A, B, and C, respectively. Positive (red) effect sizes indicate taxa with higher relative abundance in each NTM subgroup compared to those in the HC group, while negative (blue) effect sizes indicate taxa with lower relative abundance in the NTM subgroups relative to the HC group. Subgroup A refers to individuals with no prior NTM treatment who initiated antibiotic therapy, Subgroup B represents individuals currently undergoing antibiotic treatment, and Subgroup C represents individuals with a treatment history who are about to restart antibiotic therapy. HC, healthy controls; NTM, nontuberculous mycobacteria.

To identify bacterial taxa with different relative abundances between the NTM subgroups and the HC group, we conducted an analysis using the effect size derived from ALDEx2. The effect size quantifies the magnitude of an effect independent of the sample size, making it a useful approach for identifying taxa’s differences even in groups with small sample sizes. In this analysis, positive effect sizes indicate taxa that are higher than those in the HC group, while negative values indicate taxa that are lower than those in the HC group. For each NTM subgroup, the 10 most abundant and 10 least abundant taxa compared to those in the HC group, based on the highest absolute values of effect size, are shown in Figure 3. When comparing results across the NTM subgroups, certain taxa were found to be common (Table 3).

## 4. Discussion

In this study, we explored differences in gut microbiota between Japanese female patients with NTM-PD and HCs, focusing on diversity, treatment-related effects, and microbial signatures associated with disease susceptibility. Although no significant age differences were observed, the lower BMI in the NTM group aligned with previous reports of lean body habitus as a risk factor for NTM-PD [11].

When comparing bacterial taxa with reduced relative abundance compared to that in the HC group, as shown in Figure 3, a notable difference was observed between Subgroup A (pre-treatment) and Subgroup B (during treatment).

The α-diversity of the gut microbiota in Subgroup C, which was at a pre-treatment stage despite a history of recurrence, was lower than that in Subgroup A but tended to be higher than that in Subgroup B. Additionally, results for the β-diversity (Figure 1) suggested that Subgroup C had partially recovered from the disruption in gut microbiota diversity caused by previous treatments. However, interestingly, nine bacterial taxa in Subgroups C and B showed common trends in the increase and decrease in relative abundance, compared to those in the HC group (Table 3). This suggests that the dysbiotic composition of the gut microbiota may persist even after treatment. It is possible that normal resident gut bacteria are difficult to reacquire once lost, and that treatment could lead to the stabilization of dysbiotic gut microbiota that predispose individuals to NTM-PD.

In addition, nine bacterial taxa in both pre-treatment Subgroups A and C exhibited common trends in the increase and decrease in relative abundance, compared to those in the HC group (Table 3). The relative abundance of these taxa is shown in Supplementary Figure S1. These taxa may be associated with susceptibility to NTM-PD.

The bacterial taxa *Sutterella*, *Adlercreutzia*, *Odoribacter*, and *Prevotella* were commonly found to have lower relative abundance in both Subgroups A and C than in the HC group. Interestingly, *Sutterella* may play a role in immune regulation by stimulating the immune system through attachment to intestinal epithelial cells. Therefore, the depletion of *Sutterella* may impact the immune response needed to prevent NTM infection. *Adlercreutzia* has been reported to produce equol [26] and exhibit anti-inflammatory properties [27]. Equol, a compound derived from isoflavones, has strong estrogenic activity [28,29,30]. It is well known that lean, older female patients are more prone to NTM-PD, and a hypothesis has been proposed that a decrease in estrogen levels is one of the factors contributing to increased susceptibility to NTM-PD [10]. The depletion of *Adlercreutzia* may reduce the opportunity to benefit from the estrogen-reducing effect of equol, potentially increasing susceptibility to NTM-PD. *Odoribacter* is a short-chain, fatty acid-producing bacterium that is thought to establish beneficial interactions with the human host. Moreover, the outer membrane vesicles of *Odoribacter splanchnicus* have reportedly shown potential immunomodulatory effects [31]. A decrease in the abundance of *P. copri* has been significantly associated with NTM-PD and disease severity [11]. Furthermore, in mice treated with antibiotics, oral administration of *P. copri* or its capsular polysaccharides enhanced TLR2 signaling, restored immune responses, and reduced susceptibility to NTM-PD. According to these findings, the depletion of these four bacterial taxa in the gut microbiota may increase susceptibility to NTM-PD.

The bacterial taxa that were more abundant than those in the HC group and common between Subgroups A and C included *Erysipelatoclostridium*, *Massilimicrobiota*, *Flavonifractor*, *Eggerthella*, and *Fusobacterium*. It should be noted that the classification of *Erysipelatoclostridium* in this study is based on the RDP database, and its valid taxonomic name is *Thomasclavelia* [32]. *Erysipelatoclostridium*, *Flavonifractor*, and *Eggerthella* were also observed to have higher relative abundances in the gut microbiota of patients with mild cognitive impairment (MCI) than in the gut microbiota of HCs, in a report by Hatayama et al.; this suggested microbiome dysbiosis in patients with MCI [16]. The combination of increased *Erysipelatoclostridium*, which produces IgA protease [33], *Eggerthella*, which oxidizes bile acids [34], and *Flavonifractor* may contribute to the dysregulation of gut microbiota and potentially maintain a state of dysbiosis, as suggested in the discussion by Hatayama et al. [16]. In a study conducted in South Korea, *Erysipelatoclostridium ramosum* was more common in the NTM patient group than in the HC group [12]. It is possible that the increased abundance of *Erysipelatoclostridium* in individuals with NTM, not only in Japanese populations, may play an important role. *Eggerthella* strains are known to have equol-producing capabilities [30,35,36]. The increase in *Eggerthella* may counterbalance the depletion of *Adlercreutzia* in terms of equol production. However, because the equol production ability varies by strain, this remains speculative according to the genus-level information available in this study. Regarding equol, it is necessary to measure actual production levels in individuals; however, this could not be achieved in the present study, making further discussion difficult. *Massilimicrobiota* have been reported to be prevalent in the gut microbiota of Japanese female patients with depression, although most of their characteristics remain unclear [37,38]. *Fusobacterium* is a common member of both the gut and oral microbiota [39]. It is possible that certain oral abnormalities could have led to an increase in *Fusobacterium* in the intestines of individuals with NTM-PD.

Importantly, our findings suggest that antibiotic therapy may not only exacerbate dysbiosis but also stabilize an altered microbial state that could predispose patients to recurrent disease. Given the difficulty in restoring lost microbial taxa, interventions such as oral *P. copri*-derived components or targeted prebiotics supporting key taxa like *Sutterella*, which primarily inhabits the duodenum, may offer promising avenues. While FMT could be considered, its efficacy may be limited to colonic taxa and may be inadequate for organisms dominant in the upper gastrointestinal tract [40].

These observations support the idea that maintaining a healthy gut microbiota may help prevent NTM-PD. Lifestyle, diet, and routine microbial monitoring could play preventive roles in high-risk populations.

This study has some limitations. First, we could not control for confounders such as BMI, diet, and environment. Second, the findings may not be generalizable beyond the Japanese female population. Third, gut microbiota differs by sex, and our results may not apply to males. Lastly, the small sample size limits the statistical power, warranting validation in larger cohorts.

Nonetheless, this study identifies characteristic dysbiosis in Japanese female patients with NTM-PD, shaped in part by antibiotic exposure. The persistence of such dysbiosis after treatment highlights the need for novel microbiota-targeted preventive and therapeutic strategies.

## 5. Conclusions

This study revealed that Japanese female patients with NTM-PD aged 50 to 80 years exhibit gut microbiota dysbiosis even before treatment initiation, and that this dysbiosis is further exacerbated and potentially stabilized by antibiotic therapy. Such alterations in the gut microbiota may increase susceptibility to NTM-PD, complicate treatment, and contribute to recurrence or reinfection.

These findings suggest that restoring and maintaining a healthy gut microbiota may be a key component of improving NTM-PD outcomes. Future research should focus on developing microbiota-targeted interventions, such as prebiotics, probiotics, or bacteria with potential immune-modulating effects (e.g., *P. copri*), which may support host defense mechanisms. Additionally, longitudinal and multi-ethnic studies are warranted to confirm these associations and to inform personalized therapeutic strategies for preventing and managing NTM-PD.

## Figures and Tables

**Table 1:** Age and BMI of female patients in the NTM and control groups.

	NTM (*n* = 20)	HC (*n* = 20)	*p*-Value
Age (years)	67.9 ± 8.4	67.6 ± 8.2	0.745
BMI (kg/m^2^)	18.1 ± 1.9	21.0 ± 2.9	<0.001

The data are presented as mean ± standard deviation. The *p*-values were obtained using the Wilcoxon rank-sum test. BMI, body mass index; NTM, nontuberculous mycobacteria; HC, healthy control.

**Table 2:** Characteristics of patients in the NTM subgroups.

	NTM Subgroups		
	A (*n* = 6)	B (*n* = 10)	C (*n* = 4)
During NTM treatment	No	Yes	No
Previous treatment	No	Yes or No	Yes
Use of antibiotics within 3 months	No	Yes	No
NTM pathogens (*n*)			
*Mycobacterium avium*	3	5	2
*Mycobacterium intracellulare*	2	1	1
*Mycobacterium avium* and *Mycobacterium intracellulare*	0	2	1
*Mycobacterium abscesuss*	0	1	0
Not identified	1	1	0
Age (years)	65.7 ± 8.0	68.5 ± 9.4	69.5 ± 2.6
BMI (kg/m^2^)	17.1 ± 2.0	18.0 ± 1.4	19.7 ± 1.6

Subgroup A refers to individuals with no prior NTM treatment who initiated antibiotic therapy, Subgroup B represents individuals currently undergoing antibiotic treatment, and Subgroup C represents individuals with a treatment history who are about to restart antibiotic therapy. NTM, nontuberculous mycobacteria; BMI, body mass index.

**Table 3:** Bacterial taxa with common trends of relative abundance changes between NTM subgroups, relative to those in the HC group.

	Subgroup A and B	Subgroup B and C	Subgroup A and C
Taxa with higher relative abundance	*Erysipelatoclostridium*	*Erysipelatoclostridium*	*Erysipelatoclostridium*
	*Massilimicrobiota*	*Massilimicrobiota*	*Massilimicrobiota*
	*Flavonifractor*	*Flavonifractor*	*Flavonifractor*
	*Enterococcus*	*Streptococcus*	*Eggerthella*
		*Bacteroides*	*Fusobacterium*
Taxa with lower relative abundance	*Sutterella*	*Sutterella*	*Sutterella*
	*Faecalibacillus*	*Bamesiella*	*Adlercreutzia*
		*Collinsella*	*Odoribacter*
		*Butyricimonas*	*Prevotella*

Subgroup A refers to individuals with no prior NTM treatment who initiated antibiotic therapy, Subgroup B represents individuals currently undergoing antibiotic treatment, and Subgroup C represents individuals with a treatment history who are about to restart antibiotic therapy. HC, healthy controls; NTM, nontuberculous mycobacteria.

## Data Availability

The data presented in this study are available on request from the corresponding author. The data are not publicly available due to privacy concerns.

## Supplementary Materials

The following supporting information can be downloaded at: https://www.mdpi.com/article/10.3390/biomedicines13051264/s1, Table S1: Medical history of patients with NTM-PD; Table S2: Antibiotics used for treatment of NTM subgroup B; Figure S1: A jitter plot of the relative abundance of each group of gut microbiota taxa that showed common trends in relative abundance changes between Subgroup A and Subgroup C.

## Abbreviations

The following abbreviations are used in this manuscript:

ALDEx2	ANOVA-like differential expression tool
BMI	Body mass index
FMT	Fecal Microbiota Transplantation
HC	Healthy control
NTM	Nontuberculous mycobacteria
NTM-LD	Nontuberculous mycobacterial lung disease
NTM-PD	Nontuberculous mycobacterial pulmonary disease NTM-PD
NMDS	Nonmetric multidimensional scaling
PERMANOVA	Permutational Multivariate Analysis of Variance
PERMDISP	Permutational Multivariate Analysis of Dispersion
TLR2	Toll-like receptor 2
