# Supplementary material for: Gut Microbiota Dysbiosis in Japanese Female Patients with Nontuberculous Mycobacteria-Associated Lung Disease: An Observational Study
Source: Biomedicines. 2025 May 21;13(5):1264. doi: 10.3390/biomedicines13051264 (PMC12108648; doi:10.3390/biomedicines13051264)
Supplement: Supplementary file 1 [file biomedicines-13-01264-s001.zip › biomedicines-3582954-supplementary.pdf]

biomedicines

MDPI

Supplementary Table S1. Medical history of patients with NTM-PD.

Medical history	NTM subgroup		
	A	B	C
Non	1	0	2
Bronchiectasis	0	3	0
Bronchial asthma	0	1	0
Organized pneumonia	0	1	0
Allergic rhinitis	1	0	0
Sleep apnea syndrome	0	1	0
Ovarian cyst	1	0	0
Ovarian tumor	0	1	0
Adenomyosis	1	0	0
Uterine fibroids	0	1	0
Hepatic hemangioma	1	0	0
Pulmonary hypertension	0	1	0
Post-surgery for breast cancer	0	1	1
Ureteral cancer	0	1	0
Colon cancer	0	1	0
Thyroid cancer	0	0	1
Chronic thromboembolic pulmonary hypertension	0	1	0
Chronic heart failure	1	0	0
Angina pectoris	0	0	1
Sick sinus syndrome	1	0	0
Hypertension	2	1	0
Lower limb varicose veins	1	0	0
Dyslipidemia	0	1	0
Hyperlipidemia	1	0	0
Impaired glucose tolerance	0	0	1
Diabetes mellitus	0	1	0
Cholelithiasis	0	1	0
Benign paroxysmal positional vertigo	0	1	0
Glaucoma	1	2	0
Spinal canal stenosis	0	1	0
Lumbar spinal canal stenosis	0	0	1
Sjögren’s syndrome	1	0	0
Rheumatoid arthritis	0	1	0
Gastric ulcer	0	1	0
Bilateral wrist arthritis	0	1	0
Osteoporosis	0	0	1
GERD	0	0	1
Parkinson’s disease	1	0	0

A: Treatment-naïve patients before initiating antibiotic therapy; B: Patients currently undergoing antibiotic therapy; C: Patients previously treated and scheduled to restart therapy due to recurrence.

NTM-PD: Nontuberculous Mycobacterial Pulmonary Disease
GERD: Gastroesophageal Reflux Disease

Supplementary Table S2. Antibiotics used for treatment of NTM subgroup B.

Antibiotics	Number of participants
Rifampicin, Ethambutol, Clarithromycin	5
Rifampicin, Clarithromycin	1
Ethambutol, Clarithromycin	1
Rifampicin, Clarithromycin, Levofloxacin	1
Faropenem Sodium Hydrate, Sitafloxacin, Clarithromycin	1
Erythromycin	1

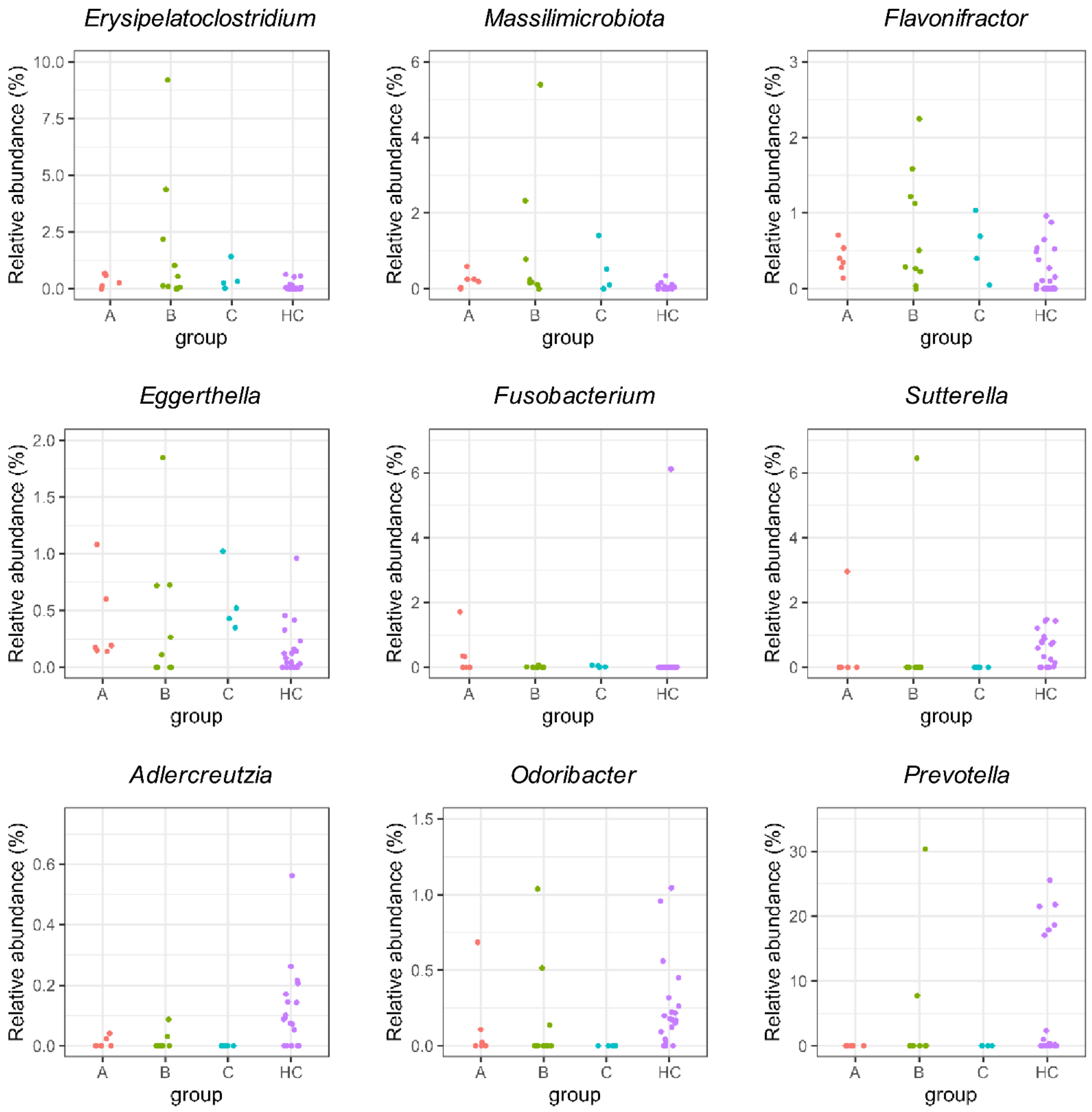

Figure S1: A jitter plot of the relative abundance of each group of gut microbiota taxa that showed common trends in relative abundance changes between Subgroup A and Subgroup C.
